# The Roles of sPLA_2_s in Skin Homeostasis and Disease

**DOI:** 10.3390/biom13040668

**Published:** 2023-04-12

**Authors:** Kei Yamamoto, Haruka Hakoi, Saki Nomura, Makoto Murakami

**Affiliations:** 1 Graduate School of Technology, Industrial and Social Sciences, Tokushima University, 2-1 Minami-jyosanjima, Tokushima 770-8513, Japan; kei@tokushima-u.ac.jp (K.Y.);; 2 Laboratory of Microenvironmental and Metabolic Health Sciences, Center for Disease Biology and Integrative Medicine, Graduate School of Medicine, The University of Tokyo (UTokyo), 7-3-1 Hongo, Bunkyo-ku, Tokyo 113-8655, Japan

**Keywords:** contact hypersensitivity, dendritic cell, epidermis, gut microbiota, hair follicle, keratinocyte, lipid metabolism, phospholipase A_2_, psoriasis, skin

## Abstract

Among the phospholipase A_2_ (PLA_2_) family, the secreted PLA_2_ (sPLA_2_) family in mammals contains 11 members that exhibit unique tissue or cellular distributions and enzymatic properties. Current studies using knockout and/or transgenic mice for a nearly full set of sPLA_2_s, in combination with comprehensive lipidomics, have revealed the diverse pathophysiological roles of sPLA_2_s in various biological events. Individual sPLA_2_s exert specific functions within tissue microenvironments, likely through the hydrolysis of extracellular phospholipids. Lipids are an essential biological component for skin homeostasis, and disturbance of lipid metabolism by deletion or overexpression of lipid-metabolizing enzymes or lipid-sensing receptors often leads to skin abnormalities that are easily visible on the outside. Over the past decades, our studies using knockout and transgenic mice for various sPLA_2_s have uncovered several new aspects of these enzymes as modulators of skin homeostasis and disease. This article summarizes the roles of several sPLA_2_s in skin pathophysiology, providing additional insight into the research fields of sPLA_2_s, lipids, and skin biology.

## 1. Introduction

**Figure 1:**
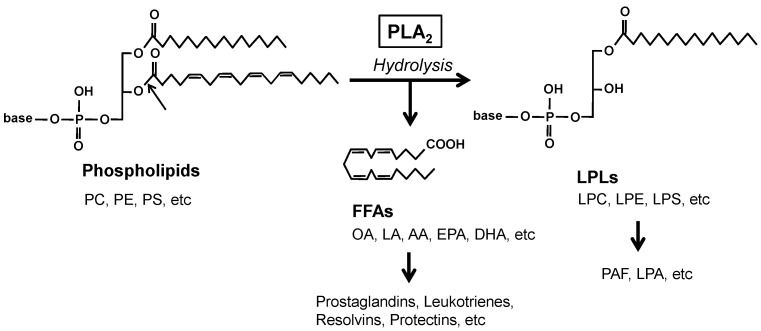
Phospholipase A_2_ (PLA_2_) reaction. PLA_2_ enzymes hydrolyze the *sn*-2 position of phospholipids (arrow) to generate free fatty acids (FFAs) and lysophospholipids (LPLs). AA, arachidonic acid; DHA, docosahexaenoic acid; EPA, eicosapentaenoic acid; LA, linoleic acid; LPA, lysophosphatidic acid; LPC, lysophosphatidylcholine; LPE, lysophosphatidylethanolamine; LPS, lysophosphatidylserine; OA, oleic acid; PAF, platelet-activating factor; PC, phosphatidylcholine; PE, phosphatidylethanolamine; PS, phosphatidylserine.

Phospholipase A_2_ (PLA_2_) enzymes hydrolyze the *sn*-2 position of glycerophospholipids (hereafter phospholipids) to generate free fatty acids (FFAs) and lysophospholipids (LPLs) (Figure 1). The mammalian genome encodes more than 50 PLA_2_s or related enzymes, which are classified into several families based on their structures and functions [1]. The PLA_2_ reaction is important for the production of lipid mediators since polyunsaturated fatty acids (PUFAs) and LPLs released by PLA_2_s can be converted to a wide variety of bioactive lipids referred to as lipid mediators, such as prostaglandins, leukotrienes, resolvins, and platelet-activating factors. In addition, PLA_2_s can also be involved in membrane remodeling by altering phospholipid composition, energy production by supplying FFAs as β-oxidation substrates, or regulation of microenvironmental lipid milieu by fine-tuning the balance between saturated and unsaturated FFAs. Moreover, several PLA_2_ enzymes catalyze non-PLA_2_ reactions, such as phospholipase A_1_, phospholipase B, lysophospholipase, triglyceride lipase, and transacylase reactions. As such, these hydrolytic actions of PLA_2_s on a wide variety of lipids are associated with various pathophysiological events, ranging from the maintenance of tissue homeostasis to inflammatory, immunological, metabolic, cardiovascular, reproductive, neurodegenerative, and oncogenic disorders.

In contrast to intracellular PLA_2_s, secreted PLA_2_s (sPLA_2_) are ideally positioned to cleave phospholipids available on the cell surface or in the extracellular milieu [2]. The sPLA_2_ family in mammals contains 11 members, namely IB, IIA, IIC (present in mice and rats, but pseudogene in humans), IID, IIE, IIF, III, V, X, XIIA, and XIIB (catalytically inactive), according to their sequence homology as well as the number and position of disulfide bonds [2]. Historically, sPLA_2_-IB and -IIA are two classical sPLA_2_s originally identified by protein purification in the late 1980s. While sPLA_2_-IB is secreted from the pancreas into the intestinal lumen and acts as a digestive enzyme [3], sPLA_2_-IIA, initially identified in the synovial fluid of arthritis patients and in platelets, is the only sPLA_2_ that is abundantly detected in the circulation of patients with inflammation or infection and has been considered to participate in systemic or local inflammation and antibacterial defense [4]. sPLA_2_-IIC and -V were identified by genomic sequencing of the locus close to the sPLA_2_-IIA (*Pla2g2a*) gene in 1994 [5,6,7,8,9]. Soon afterward, from 1997 to the early 2000s, sPLA_2_-IID, -IIE, -IIF, -III, and -X, as well as two sPLA_2_-XII isoforms, were identified using EST database searches [10,11,12,13,14,15,16,17,18,19]. Group I/II/V/X sPLA_2_s are structurally related, low-molecular-mass enzymes with a conserved His-Asp catalytic dyad, a Ca^2+^-binding loop, and 6–8 disulfide bonds that ensure strict Ca^2+^-dependent PLA_2_ reaction and protein stability, while group III and XII sPLA_2_s are each structurally atypical, having homology with group I/II/V/X sPLA_2_s only in short stretches of the catalytic and Ca^2+^-binding sites. Individual sPLA_2_s exhibit unique tissue and cellular distributions and exert specific functions in a lipid mediator-dependent or, possibly, -independent manner. In general, individual sPLA_2_s exert their specific functions within tissue microenvironments in which they are locally expressed. Although the activity of sPLA_2_s on mammalian cells is relatively weak, they can act in a paracrine fashion on the plasma membrane of activated, damaged, or dying cells in preference to resting cells [20,21,22]. More importantly, non-cellular phospholipid components, such as dietary lipids, lipoproteins, lung surfactants, extracellular vesicles (EVs), and membranes of invading microorganisms, such as bacteria and possibly fungi and parasites, act as excellent hydrolytic targets of sPLA_2_s [23,24,25,26,27,28]. In some cases, sPLA_2_-binding proteins such as PLA2R1 (sPLA_2_ receptor) modulate the functions of sPLA_2_ [29,30]. Various pathophysiological roles of individual sPLA_2_s, as demonstrated by studies using sPLA_2_ gene-manipulated (transgenic or knockout) mice over the past decades, are summarized in our current reviews [31,32,33,34,35,36]. Recently, our group has uncovered novel aspects of sPLA_2_s in skin homeostasis and diseases. This review summarizes the roles of sPLA_2_s spatiotemporally expressed in distinct cells in the context of skin homeostasis, inflammation, and cancer, thus expanding our understanding of the biological roles of sPLA_2_-driven lipid metabolism.

## 2. The Roles of Lipids in the Skin

The skin, an organ that interfaces between the host and the external environment, serves not only as a mechanical barrier to prevent water loss and the entry of harmful environmental substances and microorganisms but also as an active barrier that acts as the first line of immune defense against infection [37,38,39]. The epidermis is a highly organized epithelial tissue composed of four distinctive layers: the innermost stratum basale, stratum spinosum, stratum granulosum, and outermost stratum corneum. The dermis, a layer of skin between the epidermis and subcutaneous tissues, consists primarily of dense irregular connective tissue and cushions the body from stress and strain. Dermal fibroblasts interact with keratinocytes by secreting various growth factors and cytokines, which contribute to skin homeostasis. The hair follicle, a skin appendage formed by interactions between epidermal keratinocytes committed to hair follicle differentiation and dermal fibroblasts committed to the formation of the dermal papilla, undergoes repeated hair cycles. Additionally, immune cells being resident in normal skin or those infiltrating into inflamed skin have various effects on epidermal keratinocytes and dermal fibroblasts via the production of cytokines, chemokines, and likely bioactive lipids.

Lipids represent an essential biological component for skin homeostasis and diseases. Perturbed lipid metabolism often leads to skin abnormalities that are outwardly visible. The epidermis acts as a permeability barrier, which prevents the entry of pathogens, allergens, and harmful substances from outside the body and the loss of water and electrolytes from inside the body. The stratum corneum contains a multilayered lipid structure called lipid lamellae, whose hydrophobicity prevents the permeation of the hydrophilic materials. Lipid lamellae consist mainly of ceramides, cholesterol, and FFAs. Epidermal ceramides are composed of diverse molecular species containing ω-*O*-acylceramides, a unique class of ceramides specialized for epidermal barrier formation. Unlike normal ceramides that have a long-chain base and a fatty acyl chain of C16–24, ω-*O*-acylceramides have a very long fatty acyl chain (C28–36) that is additionally esterified with a linoleic acid (LA), being one of the most hydrophobic lipids in mammalian bodies [40,41,42]. The formation of ω-*O*-acylceramides from normal ceramides is catalyzed sequentially with the fatty acid elongase ELOVL4, the fatty acid ω-hydroxylase CYP4F22 (in humans) or CYP4F39 (in mice), and the patatin-like phospholipase PNPLA1 [42,43,44]. A pool of ω-*O*-acylceramides is further converted to protein-bound ceramides forming the cornified lipid envelope by the epidermal lipoxygenases 12R-LOX and eLOX3 followed by SDR9C7, a member of the dehydrogenase/reductase family [45,46]. The nutritional insufficiency of essential fatty acids, especially LA, impairs ω-*O*-acylceramide synthesis and ultimately causes skin abnormalities [47] and genetic mutations in multiple steps of skin lipid metabolism variably and often severely affect epidermal barrier function or hair cycling, thereby triggering or exacerbating skin disorders such as ichthyosis, psoriasis, atopic dermatitis, and alopecia [48,49,50,51]. FFAs have also been implicated in epidermal acidification for skin barrier formation [52,53,54]. Furthermore, dysregulated production of lipid mediators derived from FFAs or LPLs can be linked to various skin disorders such as hair loss, epidermal hyperplasia, dermatitis, and cancer [49,55,56]. For instance, arachidonic acid (AA)-oxygenated lipid mediators, including prostaglandins and leukotrienes, whose production is in many cases regulated by cytosolic PLA_2_α (cPLA_2_α; PLA2G4A) coupled with cyclooxygenases and lipoxygenases [57], variably regulate immunological responses in skin diseases [58]; *N*-acylethanolamines, a distinct class of lipid mediators having a structure of FFA condensed with ethanolamine, are produced by cPLA_2_ε (PLA2G4E) in epidermal keratinocytes and suppress psoriatic inflammation [59]; and lysophosphatidic acid (LPA), spatiotemporally generated by phosphatidic acid-specific phospholipase A_1_ (PA-PLA_1_α) in hair follicles, regulates hair growth and quality [55].

It has been proposed that sPLA_2_ in the skin provides FFAs that maintain the acidity of the stratum corneum [60]. Mice transgenically overexpressing human sPLA_2_-IIA (*PLA2G2A*-TG) or sPLA_2_-X (*PLA2G10*-TG) show striking skin abnormalities characterized by epidermal thickening, sebaceous gland hyperplasia, and alopecia, independently of inflammation [61,62]. Additionally, skin-specific transgenic mice for mouse sPLA_2_-IIA (*Pla2g2a*-TG) show increased skin carcinogenesis [63]. However, since neither sPLA_2_-IIA nor -X is endogenously detectable in mouse skin at a substantial level, the pathophysiological significance of the skin phenotypes observed in these transgenic mice is unclear, and it is possible that the overexpressed sPLA_2_-IIA or -X might mimic the function of other sPLA_2_(s) intrinsically expressed in mouse skin. In humans, expression of sPLA_2_-IIA and -X are detected in transformed keratinocytes, and *PLA2G2A* knockdown in human skin squamous cell carcinoma reduces tumorigenicity in a mouse xenograft model [64], pointing to the potential contribution of sPLA_2_-IIA or -X to skin cancer. The major sPLA_2_s expressed endogenously in mouse skin are sPLA_2_-IIF and sPLA_2_-IIE, the former being expressed in the epidermis [65] and the latter in hair follicles depending on the hair cycle [66]. This suggests that these two sPLA_2_s have distinctive and non-redundant roles at different sites within the skin. Moreover, sPLA_2_s expressed in immune cells and even in distal tissues can also affect skin pathology. In the following sections, the functions of several sPLA_2_s that exhibit distinct tissue/cellular localizations in skin homeostasis and disease are discussed.

## 3. sPLA_2_ in the Epidermis

The epidermis, as described above, is a highly organized stratified epithelium with distinctive keratinocyte layers. Notably, sPLA_2_-IIF is the major sPLA_2_ expressed in the suprabasal layers of mouse and human epidermis [65]. Under the basal state, *Pla2g2f*^−/−^ mice exhibit only mild skin abnormalities, characterized by a fragile stratum corneum with modest perturbation of skin barrier function and acidity. These phenotypes are more pronounced in the abdominal skin of an adult, but not neonatal, *Pla2g2f*^−/−^ mice, suggesting that although sPLA_2_-IIF is not a major player in the central program of epidermal differentiation, it contributes to increasing stratum corneum stability against environmental stresses such as friction against the floor or prolonged exposure to skin microbiota. After tape-stripping of the stratum corneum, *Pla2g2f*^−/−^ mice display delayed recovery from the skin barrier damage, suggesting that sPLA_2_-IIF accelerates epidermal repair [60]. The impact of sPLA_2_-IIF ablation is more dramatic in primary keratinocytes in culture, where the cells fail to be properly differentiated and activated when sPLA_2_-IIF is genetically ablated or pharmacologically inactivated by a pan-sPLA_2_ inhibitor [65]. Moreover, by employing an epidermal three-dimensional (3D) culture model with a human epidermal keratinocyte cell line (NHEK-SVTERT3-5), which had been immortalized by retroviral transfection of SV40 [67], the expression of *PLA2G2F* mRNA was markedly increased in accordance with upward proliferation and differentiation of keratinocyte layers, and its knockdown resulted in a decrease in the expression of keratinocyte differentiation markers and an increase in transepidermal water loss, indicative of disturbed skin barrier function (manuscript in preparation). Thus, loss of sPLA_2_-IIF impairs proper keratinocyte differentiation and barrier formation in both mice and humans.

Global or skin-specific transgenic mice overexpressing mouse sPLA_2_-IIF (*Pla2g2f*-TG) spontaneously develop psoriasis-like epidermal hyperplasia and alopecia, with increased expression of a panel of psoriasis markers, including S100A9 and IL-36α [65]. Moreover, sPLA_2_-IIF is induced in mouse skin treated with imiquimod, an inducer of experimental psoriasis, and is also highly expressed in the hyperplasic epidermis of patients with psoriasis. Importantly, genetic deletion of sPLA_2_-IIF in mice protects against epidermal hyperplasia and associated inflammation in models of Th17-dependent psoriasis, Th1-dependent contact hypersensitivity (CHS), and skin carcinogenesis. These findings indicate that sPLA_2_-IIF is associated with the exacerbation of epidermal-hyperplasic diseases. Mechanistically, sPLA_2_-IIF preferentially hydrolyzes plasmalogen (alkenyl-type phosphatidylethanolamine (PE)) having an *sn*-2 PUFA (DHA in particular) secreted from keratinocytes to yield lysoplasmalogen (P-LPE). This unique LPL facilitates aberrant proliferation and activation of keratinocytes, leading to the propagation of skin inflammation. Indeed, the levels of P-LPE in mouse skin are correlated well with the expression levels of sPLA_2_-IIF in multiple skin disease models, and topical application of P-LPE to *Pla2g2f*^−/−^ skin in vivo or supplementation of *Pla2g2f*^−/−^ keratinocytes with P-LPE ex vivo restores the psoriasis-related phenotypes [65].

Thus, the sPLA_2_-IIF/P-LPE axis has beneficial and detrimental roles in skin barrier formation and epidermal-hyperplasic inflammation, respectively, thereby regulating the physiology and pathology of the skin. Since LPLs with an *sn*-1 alkenyl moiety are structurally unstable and can be readily degraded non-enzymatically under acidic conditions, it is plausible that P-LPE exists more stably in inflamed skin where epidermal pH becomes close to neutral. In contrast, in healthy skin where epidermal pH is mildly acidic, P-LPE might be further converted to a certain stable metabolite that regulates skin homeostasis and repair, a possibility that is now under investigation.

## 4. sPLA_2_ in Hair Follicles

Abnormalities in skin lipid metabolism vary and often severely affect hair cycling, causing hair loss or alopecia [49,55]. Hair follicles in the skin undergo repeated cycles of growth (anagen), regression (catagen), and rest (telogen) during life [68]. sPLA_2_-IIE is abundantly expressed in hair follicles during the anagen period, being distributed in companion cells of the outer root sheath and cuticular cells of the inner root sheath [66]. In *Pla2g2e*^−/−^ mice, hair follicles show a detachment between the follicular epithelium (cuticle) and hair shaft and altered expression of some hair follicle-related genes, but with little or no abnormalities in the epidermis. Lipidomics analysis has revealed that sPLA_2_-IIE mobilizes various unsaturated FFAs and LPE species in mouse skin, consistent with the in vitro substrate specificity of sPLA_2_-IIE. However, it remains unclear which lipid metabolites mobilized by sPLA_2_-IIE participate in hair follicle homeostasis and whether sPLA_2_-IIE also plays a similar role in hair quality control in human skin.

## 5. sPLA_2_ in Lymphoid Tissues That Affects Skin Diseases by Regulating Adaptive Immune Responses

While sPLA_2_-IIE and sPLA_2_-IIF are abundantly expressed in keratinocytes of the upper epidermis and hair follicles, respectively (see above), sPLA_2_-IID is barely detectable in mouse skin. Instead, sPLA_2_-IID is expressed abundantly in dendritic cells (DCs) and macrophages, especially CD4^+^CD11b^+^CD11c^+^ MHC class II^lo^ DCs and M2-like macrophages, in secondary lymphoid organs such as the spleen and lymph nodes (LNs) of mice and humans [69,70]. Furthermore, sPLA_2_-IID expression is downregulated, rather than upregulated, in DCs stimulated with antigen or lipopolysaccharide [69,71]. A lipidomics-based PLA_2_ enzyme assay using a natural phospholipid mixture extracted from mouse lymphoid tissue as a substrate [72] indicates that sPLA_2_-IID preferentially hydrolyzes PE species with an *sn*-2 PUFA, including ω6 AA and, more efficiently, ω3 eicosapentaenoic acid (EPA) and docosahexaenoic acid (DHA), rather than those with oleic acid and LA. This enzymatic preference of sPLA_2_-IID for PE species with an ω3 PUFA as substrates, along with its distribution in lymphoid immune cells and downregulation by proinflammatory stimuli, suggests that sPLA_2_-IID has a resolving, rather than promoting, role in the adaptive immune response. In fact, despite the low expression of sPLA_2_-IID in the skin, *Pla2g2d* deficiency leads to exacerbation of CHS and psoriasis, likely because sPLA_2_-IID attenuates adaptive immunity in the LNs, thereby sequestering pathogenic Th1 and Th17 immune responses [69,70].

In a model of Th1-dependent CHS, topical application of the hapten antigen dinitrofluorobenzene to abdominal skin (sensitization), followed by a second application of the same antigen to ear skin (elicitation), induces ear swelling. In the elicitation phase of CHS, the resolution of inflammation in the skin and LNs is delayed in *Pla2g2d*^−/−^ mice [69]. In this state, expression levels of the Th1 cytokines IFN-γ and IL-12 are greater in the draining LNs of *Pla2g2d*^−/−^ mice than in those of littermate wild-type (WT) mice. Likewise, in a model of psoriasis, *Pla2g2d*^−/−^ mice display more severe epidermal hyperplasia than do WT mice, with increased IL-17A^+^ or IL-22^+^ T cells in the affected skin and LNs [70]. Furthermore, DCs isolated from *Pla2g2d*^−/−^ mice are hyperactivated even without stimulation. Mechanistically, sPLA_2_-IID in the LNs constitutively hydrolyzes PUFA-containing PE species (possibly in EV membranes) to mobilize ω3 PUFA-derived anti-inflammatory lipid mediators that put a brake on DC-mediated adaptive immunity. Indeed, steady-state levels of ω3 PUFAs and their metabolites, such as DHA-derived resolvin D1 (RvD1), are markedly reduced in LNs from *Pla2g2d*^−/−^ mice compared to WT mice. Conversely, *Pla2g2d*-TG mice display milder inflammation than do WT mice in the CHS and psoriasis models, with increased levels of ω3 PUFA metabolites [70]. ω3 PUFA-derived resolvins and maresins suppress acquired immunity by attenuating DC migration, activation, and antigen presentation to T cells and by preventing IgE class switching in B cells [69,73,74,75]. Moreover, these ω3 PUFA-derived lipid mediators can facilitate the polarization of anti-inflammatory M2 macrophages [76,77], consistent with the fact that fewer M2 macrophages are present in the LNs of *Pla2g2d*^−/−^ mice [70].

On the other hand, the beneficial role of sPLA_2_-IID in counteracting pathogenic Th1/Th17 immune responses can be conversely disadvantageous in some situations, such as host defense against infection and cancer [70,78]. Indeed, sPLA_2_-IID promotes, rather than prevents, the development of skin tumors, likely because it attenuates anti-tumor Th1 immunity. Accordingly, *Pla2g2d*^−/−^ mice are protected against skin carcinogenesis, with increased tumor-suppressing IFN-γ^+^CD8^+^ cytotoxic T cells and M1 macrophages [70]. Thus, the immunosuppressive function of sPLA_2_-IID provides “good” or “bad” outcomes in distinct disease settings, ameliorating skin inflammation and exacerbating skin cancer. sPLA_2_-IID also alleviates anti-viral immunity, possibly through mobilizing anti-inflammatory PGD_2_ in the lung, and ultimately exacerbates coronavirus-induced acute lung injury [78,79,80]. Thus, specific inhibition of sPLA_2_-IID in patients with certain types of cancer or infection could be an attractive therapeutic intervention for restoring immunological functions, a concept reminiscent of “immune checkpoint” therapy.

## 6. sPLA_2_ Involved in an Alteration of the Intestinal Microbiota That Secondarily Affects Skin Diseases

Since sPLA_2_-IIA is induced in various tissues during inflammation in humans and rats, its functions have been proposed to be related to the exacerbation of inflammation through the production of lipid mediators at the local sites of expression and to host defense against infectious bacteria through the degradation of bacterial membrane phospholipids [4]. However, the *Pla2g2a* gene is naturally disrupted in C57BL/6 and 129/Sv strains due to a frameshift mutation [81], which makes it difficult to assess the precise functions of endogenous sPLA_2_-IIA in vivo using a standard knockout strategy. Other mouse strains, such as BALB/c, C3H, and DBA/1, have an intact *Pla2g2a* gene [81], but unlike the situation in humans and rats, its expression in these mouse strains is highly restricted to the intestine [82,83]. Despite this biased distribution, genetic deletion of sPLA_2_-IIA in the BALB/c strain results in attenuation of the development of carcinogen-induced skin cancer and aggravation of imiquimod-induced psoriasis [84]. Therefore, it seems a mystery why these phenotypes are manifested in mouse skin where sPLA_2_-IIA is minimally expressed.

In the small intestine, sPLA_2_-IIA is predominantly expressed in Paneth cells that secrete a variety of antimicrobial peptides, and its expression is significantly reduced by antibiotic administration [84]. This raises the possibility that sPLA_2_-IIA may be induced by intestinal bacterial components and, as an antimicrobial protein, may have a secondary effect on the skin by degrading intestinal bacterial membranes and thereby altering the balance of the gut microbiota. A comparative analysis of the gut microbiota has revealed apparent differences in several bacterial genera (*Helicobacter*, *Ruminococcus*, *Lachnospira*, etc.) between *Pla2g2a*^−/−^ and WT mice. Furthermore, when *Pla2g2a*^−/−^ mice and WT mice are co-housed from birth, under which gut microbiota in the two groups no longer differ, the differences in skin phenotypes between the genotypes are lost. The small intestine of *Pla2g2a*^−/−^ mice shows notable changes in the expression of a group of genes involved in the epithelial barrier and immunity, especially that of immunoglobulin genes, reflecting the differences in the intestinal microbiota. Various plasma metabolites, including those involved in immune modulation and oncogenesis, are significantly altered in *Pla2g2a*^−/−^ mice compared to WT mice. A comprehensive analysis of fecal lipids has revealed a significant decrease in unique lipid metabolites that are likely to be derived from the gut bacteria rather than from the host in *Pla2g2a*^−/−^ mice. Furthermore, when housed in a cleaner animal facility, the intestinal microbiota, including *Helicobacter* and *Ruminococcus,* are reduced, the intestinal expression of sPLA_2_-IIA is decreased, and the skin phenotypes caused by sPLA_2_-IIA deficiency are mitigated. These results collectively suggest that sPLA_2_-IIA secreted from small intestinal Paneth cells is involved in the shaping of the intestinal microbiota and that when this pathway is perturbed, the intestinal microbiota are altered, blood metabolites and immune responses are changed, and the phenotypes eventually become evident in distal organs such as the skin. In further support of this conclusion, PLA2G2A-TG mice on the C57BL/6 strain also display an alteration in the gut microbiota, which leads to the exacerbation of systemic inflammation and arthritis [85].

These findings are the first to elucidate the function of sPLA_2_-IIA in the intestinal tract, which has remained unknown for many years. Since several sPLA_2_ isoforms other than sPLA_2_-IIA are also expressed in the intestinal tract, it is possible that they may also affect the pathophysiology in distant organs via regulation of the intestinal microbiota, although future work is needed to prove this hypothesis and generalize the theory. Beyond the difference in the expression profiles of sPLA_2_-IIA between humans and mice, as mentioned above, high expression of sPLA_2_-IIA in the intestine of both species suggests that this bactericidal sPLA_2_ is likely to be involved in the regulation of the intestinal microbiota in humans as well. Therefore, drug discovery targeting sPLA_2_-IIA in the intestinal tract may be useful for new diagnoses and treatment of skin diseases.

**Figure 2:**
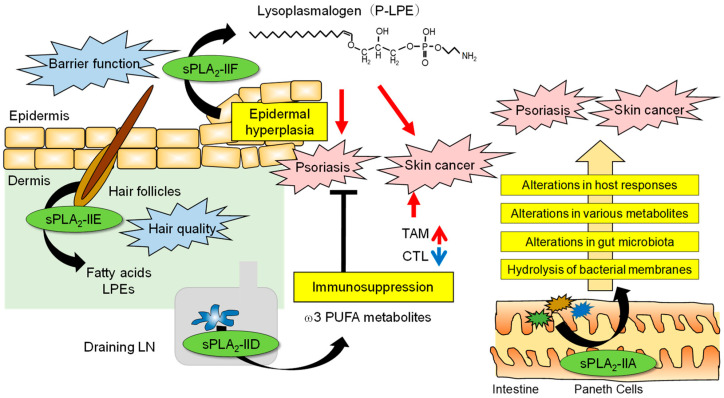
The roles of sPLA_2_s in skin homeostasis and disease. The skin is an organ that interfaces between the host and the external environment. The major sPLA_2_s expressed endogenously in mouse skin are sPLA_2_-IIF and sPLA_2_-IIE, the former in the epidermis and the latter in hair follicles, depending on the hair cycle. Following a psoriatic stimulus, sPLA_2_-IIF is induced in epidermal keratinocytes by Th17 cytokines derived from T and Th17 cells and preferentially hydrolyzes plasmalogen to give rise to P-LPE, which in turn promotes epidermal hyperplasia and inflammation. In contrast, sPLA_2_-IID blocks Th17 immunity in the draining LN through the production of ω3 PUFA metabolites, thereby putting a break on psoriasis. In the case of skin cancer, P-LPE produced by epidermal sPLA_2_-IIF promotes hypergrowth of skin cancer without affecting its incidence, while ω3 PUFA metabolites produced by sPLA_2_-IID in the LN decrease IFN-g^+^CD8^+^ cytotoxic T cells (CTLs) and increase M2-like tumor-associated macrophages (TAMs). As such, sPLA_2_-IID reduces anti-tumor immunity and ultimately facilitates tumor formation and growth. In the intestinal lumen, sPLA_2_-IIA secreted from Paneth cells acts as an antimicrobial protein to shape the gut microbiota, thereby secondarily affecting host responses, including psoriasis and skin cancer.

The roles of sPLA_2_s in skin homeostasis and disease are summarized in Figure 2.

## 7. Summary and Future Prospects

Our current studies using knockout and/or transgenic mice for nearly a full set of isozymes in the sPLA_2_ family have enabled us to conduct comparative phenotypic analysis. Under physiological conditions, sPLA_2_-IIF, mainly localized in the suprabasal layers of the epidermis, contributes to facilitating epidermal barrier function [65], while sPLA_2_-IIE, spatiotemporally expressed in hair follicles during anagen, contributes to controlling hair quality [66]. Under pathological conditions, sPLA_2_-IIF is induced in the epidermis by Th17 cytokines or possibly by other factors and plays an exacerbating role in skin disorders such as psoriasis, CHS, and skin cancer via the production of P-LPE [65]. sPLA_2_-IID, highly expressed in DCs within lymphoid tissues, constitutively mobilizes anti-inflammatory lipid mediators derived from ω3 PUFAs to counter the acquired immune responses [69,70]. Therefore, in sPLA_2_-IID-deficient mice, Th1-driven CHS and Th17-driven psoriasis are exacerbated, whereas skin cancer is attenuated because of enhanced anti-tumor immunity [84]. sPLA_2_-IIA, secreted from small intestinal Paneth cells, is involved in the shaping of the intestinal microbiota, thereby indirectly affecting cancer and psoriasis in distal skin [84].

Current indole-based sPLA_2_ inhibitors, such as indoxam and varespladib, block group I/II/V/X sPLA_2_s potently but broadly, which may limit their clinical application [86,87,88]. Apparently, the development of sPLA_2_-IIF-specific inhibitors would be desirable for the treatment of skin diseases because pan-sPLA_2_ inhibitors can also suppress the anti-inflammatory function of sPLA_2_-IID, which is expected to worsen, rather than ameliorate, inflammatory diseases. In this context, the reduced anti-tumor immunity from sPLA_2_-IID deficiency is consistent with the concept of the immune checkpoint, which has recently attracted attention in the field of cancer therapy, suggesting that sPLA_2_-IID may be a novel drug target for skin cancer. Although the oral application of mice with methyl indoxam, a pan-sPLA_2_ inhibitor, has been reported to suppress diet-induced obesity and glucose intolerance likely by inhibiting dietary phospholipid digestion with sPLA_2_-IB [89], the effect of this inhibitor on intestinal sPLA_2_-mediated regulation of the gut microflora [23,24] should also be considered. Nevertheless, translation of the results obtained from mouse models to human pathology is more complex since expression patterns of sPLA_2_ and tissue profiles of lipids are not entirely identical across animal species. While epidermal expression of sPLA_2_-IIF and DC expression of sPLA_2_-IID are well conserved between mice and humans, expression profiles of sPLA_2_-IIA and -IIE are quite distinct in both species. It should also be noted that the tissue-intrinsic effects of sPLA_2_, the extrinsic effects of sPLA_2_ from distal tissues, or a combination thereof can be variably affected by environmental factors. With these limitations in mind, our studies have revealed that multiple sPLA_2_s participate in the regulation of skin pathophysiology through different mechanisms, and accumulating knowledge on the functions of individual sPLA_2_s will hopefully lead to drug discovery for sPLA_2_-driven skin diseases.

## Data Availability

The data that support the findings of this study are available from the corresponding author, M.M., upon reasonable request.
